# Fibroblast Growth Factor-9 Activates c-Kit Progenitor Cells and Enhances Angiogenesis in the Infarcted Diabetic Heart

**DOI:** 10.1155/2016/5810908

**Published:** 2015-11-22

**Authors:** Dinender Singla, Jing Wang

**Affiliations:** Biomolecular Science Center, Burnett School of Biomedical Sciences, College of Medicine, University of Central Florida, Orlando, FL 32816, USA

## Abstract

We hypothesized that fibroblast growth factor-9 (FGF-9) would enhance angiogenesis via activating c-kit positive stem cells in the infarcted nondiabetic and diabetic heart. In brief, animals were divided into three groups: Sham, MI, and MI+FGF-9. Two weeks following MI or sham surgery, our data suggest that treatment with FGF-9 significantly diminished vascular apoptosis compared to the MI group in both C57BL/6 and db/db mice (*p* < 0.05). Additionally, the number of c-kit^+ve^/SM *α*-actin^+ve^ cells and c-kit^+ve^/CD31^+ve^ cells were greatly enhanced in the MI+FGF-9 groups relative to the MI suggesting FGF-9 enhances c-Kit cell activation and their differentiation into vascular smooth muscle cells and endothelial cells, respectively (*p* < 0.05). Histology shows that the total number of vessels were quantified for all groups and our data suggest that the FGF-9 treated groups had significantly more vessels than their MI counterparts (*p* < 0.05). Finally, echocardiographic data suggests a significant improvement in left ventricular output, as indicated by fractional shortening and ejection fraction in both nondiabetic and diabetic animals treated with FGF-9 (*p* < 0.05). Overall, our data suggests FGF-9 has the potential to attenuate vascular cell apoptosis, activate c-Kit progenitor cells, and enhance angiogenesis and neovascularization in C57BL/6 and db/db mice leading to improved cardiac function.

## 1. Introduction

Diabetes, characterized by dysregulated circulating blood glucose levels, is consequent to pancreatic beta cell destruction yielding little to no insulin production (type I, insulin-dependent diabetes mellitus, IDDM) or insulin resistance stemming from genetic predisposition, age, obesity, hypertension, and/or sedentary lifestyle habits (type II, noninsulin dependent diabetes mellitus, NIDDM). Myocardial infarction (MI), in the context of NIDDM patients, is much more prevalent compared to nondiabetic counterparts with associated increased risk of post-MI morbidity and mortality [1–3]. Consequent to MI in NIDDM patients, dynamic, complex, and adverse vascular and myocardial remodeling results in an attempt to rescue endogenous left ventricular structure and function. Such processes are characterized by (1) cell death via apoptosis and necrosis of cardiac cell types including cardiac myocytes, (2) fibroblast infiltration and scar formation, (3) hypertrophy, and (4) vascular cell death including vascular smooth muscles (VSM) and endothelial cell (EC) types [4–7]. Previous reports have suggested that abnormal myocardial angiogenesis in the setting of diabetes may be resultant of enhanced vascular cell type death and dysregulated angiogenic growth factors and cytokines such as vascular endothelial growth factor (VEGF) and angiopoietin-1 (Ang-1) [8–10]. Gene therapy has been attempted, with use of various factors including Ang-1, in diabetic animal models to promote appropriate vascular maturation and development [8–10]. Although significant improvement in impaired angiogenesis was noted, optimal gene therapy has yet to be identified in NIDDM patients and animal models and remains a major challenge.

Fibroblast growth factors (FGFs) comprise a large family of polypeptide growth factors that contribute to a host of biological functions including embryonic development, tissue morphogenesis, and physiological homeostasis [11, 12]. In particular, FGF-9, like other family members, has been shown to mediate variegated cellular processes including neuronal cell growth and development, midgestational myocardial proliferation and coronary neovasculogenesis, testicular embryogenesis, hair follicle neogenesis, inner ear morphogenesis, and joint development [13–19]. Recently, data has been published suggesting conditional transgenic FGF-9 expression in the post-MI heart enhanced microvessel density and left ventricular hypertrophy, reduced interstitial fibrosis, improved systolic function, and mitigated subsequent death [19]. However, whether FGF-9 generates neovascularization in the post-MI infarcted diabetic heart remains elusive. Within the current study, we hypothesized that transplanted FGF-9 would inhibit vessel and capillary apoptosis and activate endogenous c-Kit^+ve^ cells for their differentiation into VSM and EC types, contributing to neovascularization in the post-MI nondiabetic and diabetic heart.

## 2. Materials and Methods

### 2.1. MI and FGF-9 Administration

MI was generated in diabetic db/db and C57BL/6 mice (8–12 weeks old) as approved by the University of Central Florida Institutional Animal Care and Use Committee (IACUC) and as previously detailed [20, 21]. db/db and C57BL/6 animals were divided independently into three groups (*n* = 7–9 animals/group): sham, MI, and MI + FGF-9 (1 ng/10 *μ*L 0.1% BSA in 1X PBS). In brief, mice were anesthetized with isoflurane and a left thoracotomy was performed. The left descending coronary artery was visualized and permanently ligated. In the MI + FGF-9 group, FGF-9 was delivered via two injections (1 ng FGF-9/injection prepared as aforementioned for a total dose of 2 ng FGF-9) into the peri-infarct region using a 29-gauge floating needle. Sham operated animals underwent all surgical protocols with permanent artery ligation excluded. On day 14 post-MI, animals were sacrificed using 4% inhalatory isoflurane for 10 mins followed by cervical dislocation. Hearts were subsequently removed and preserved for further analysis.

### 2.2. Apoptotic Vascular Cell Identification

All protocols for VSM and endothelial cell immunostaining were carried out as previously described [22]. In brief, 5 *μ*m serial sections were cut at the midpapillary level from paraffin embedded heart sections. Heart sections were deparaffinized in xylene, rehydrated by sequential incubation in 100%, 95%, 70%, and 30% alcohol, and permeabilized with proteinase K (Sigma Aldrich cat # P6556-10 mg, 25 *μ*g/mL in 100 mM Tris HCl, cat # BP152-5, Fisher Scientific). Apoptotic nuclei were visualized using a TUNEL assay (TMR red, cat # 12156792910, Roche Applied Bio Sciences) as previously reported [22]. After TUNEL staining, heart sections were costained overnight with primary antibodies against anti-SM *α*-actin (vascular smooth muscle cells, 1 : 100, cat # A2172-0.2 mL, Sigma Aldrich) followed by an antimouse antibody (M.O.M. kit, cat # FMK-2201, Vector laboratories). To detect endothelial cells post-TUNEL staining, heart sections were incubated with anti-CD31 (1 : 100, cat # 250589, Abbiotec) followed by incubation with goat antirabbit IgG-FITC secondary antibodies (1 : 30, cat # sc-2012, Santa Cruz). Sections were then mounted with Vectashield (Vector Laboratories, cat # H-1200) containing DAPI and viewed under fluorescent and confocal microscopy. Left ventricular vessels within the peri-infarcted area of the left ventricle, containing colocalized SM *α*-actin and TUNEL or anti-CD31 and TUNEL, were considered apoptotic and quantified in 1-2 sections from *n* = 5-6 animals/group.

### 2.3. c-Kit Activation and Differentiation

As previously reported, sections were deparaffinized in xylene, rehydrated in alcohol, washed with distilled water and PBS, and covered with 10% normal goat serum (NGS, cat # s-1000, Vector Laboratories) for one hour to prevent nonspecific binding [22, 23]. Heart sections were incubated for one hour with mouse monoclonal primary antibodies against c-Kit (1 : 20, cat # sc-365504, Santa Cruz) and costained with anti-SM *α*-actin (1 : 15, cat # A2172-0.2 mL, Sigma Aldrich) or anti-CD31 (1 : 20, cat # sc-46694 Santa Cruz). Sections were then incubated with appropriate secondary antibodies (Alexa Fluor 568- or 488-conjugated goat antimouse IgG, cat # A11019, Invitrogen) for one hour. Following secondary antibody incubation, stained heart sections were mounted with Vectashield antifade medium (cat # H-1200, Vector Laboratories) containing DAPI and examined under confocal microscopy for photomicrographs and quantitative analysis. For quantitative analysis, four images per section (1-2 sections, *n* = 5 animals/group) within the peri-infract region of the left ventricle were used to obtain the average number of c-kit cells positive for SM *α*-actin or CD31.

### 2.4. Histological Quantification of Ventricular Vasculature

To visualize and quantify ventricular vasculature, prepared heart sections were stained with Masson's trichrome as previously described [6]. The number of vessels (in the peri-infarct region of the left ventricle) was quantified in 1-2 sections from *n* = 6–8 animals per group using an Olympus microscope at 20x magnification.

### 2.5. Cardiac Function

Left ventricular function was determined using a Phillips Sonos 5500 ultrasound system. Animals were anesthetized with isoflurane and placed in the supine position on a controlled heating pad. Using a 15–6 L hockey-stick transducer, two-dimensional images were recorded and M-mode frames were used to measure left ventricular internal dimension-diastole (LVIDd), left ventricular internal dimension-systole (LVIDs), fractional shortening (FS, [(LVIDd − LVIDs)/LVIDd] × 100), left ventricular volume at end diastole (EDV), left ventricular volume at end systole (ESV), and ejection fraction (EF, [(EDV − ESV)/EDV] × 100) in the short-axis view at the midpapillary muscle level.

### 2.6. Data Analysis

All values are presented as a mean ± SEM. Analysis of data was performed using one-way ANOVA followed by the Tukey post hoc test using SigmaStat. Statistical significance was assigned when *p* < 0.05.

## 3. Results

### 3.1. FGF-9 Prevents Vessel Apoptosis Post-MI

**Figure 1:**
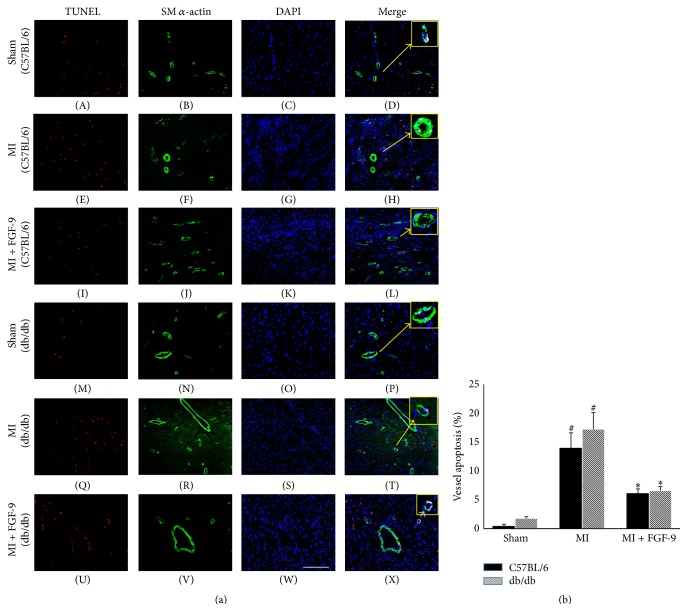
FGF-9 blunts vessel apoptosis in the diabetic infarcted heart. Representative photomicrographs illustrating vascular apoptosis are depicted in (a) for C57BL/6 (A–L) and db/db (M–X) mice with TUNEL positive nuclei in red (A, E, I, M, Q, and U), SM *α*-actin^+ve^ cells in green (B, F, J, N, R, and V), total nuclei stained blue with DAPI (C, G, K, O, S, and W), and merged images (D, H, L, P, T, and X). The boxes on the top right panel of (a) are enhanced images from each group to demonstrate the colocalization of TUNEL, SM *α*-actin, and DAPI within a single vessel. Scale bar = 100 *μ*m. (b) Histogram of quantitative vessels apoptosis in infarcted C57BL/6 and db/db mice. ^#^ *p* < 0.05 versus sham and ^*∗*^ *p* < 0.05 versus MI.

Representative photomicrographs illustrating vascular apoptosis are shown in Figure 1(a) for C57BL/6 (A–L) and db/db (M–X) mice with TUNEL positive nuclei in red (A, E, I, M, Q, and U), SM *α*-actin in green (B, F, J, N, R, and V), total nuclei stained blue with DAPI (C, G, K, O, S, and W), and merged images (D, H, L, P, T, and X). The boxes on the top right panel of Figure 1(a) are enhanced images from each group to demonstrate the colocalization of TUNEL, SM *α*-actin, and DAPI within a single vessel. Quantification of apoptotic vessels suggests vascular apoptosis is significantly enhanced in C57BL/6 and db/db mouse hearts post-MI relative to sham operated C57BL/6 and db/db mice (*p* < 0.05, Figure 1(b)). Importantly, FGF-9 treatment post-MI dramatically mitigated vessel apoptosis in both C57BL/6 and db/db mice compared to their MI operated counterparts (*p* < 0.05, Figure 1(b)). However, no statistical significance was noted between C57BL/6 and db/db vasculature apoptosis outcomes in sham, MI, or MI + FGF-9 groups (Figure 1(b)).

### 3.2. FGF-9 Reduces Capillary Apoptosis following MI

**Figure 2:**
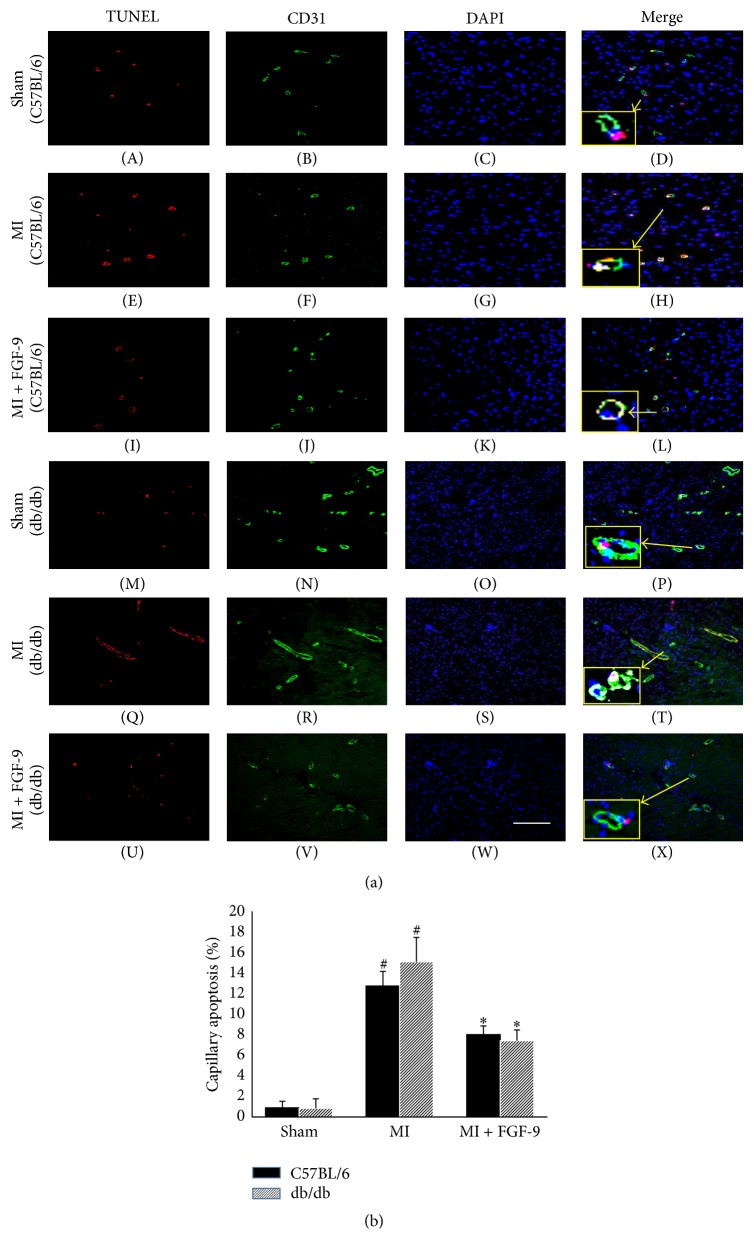
Effects of FGF-9 on capillary apoptosis following MI in C57BL/6 and db/db mice. Representative images demonstrating capillary apoptosis are illustrated in (a) for C57BL/6 (A–L) and db/db (M–X) mice with TUNEL positive nuclei in red (A, E, I, M, Q, and U), CD31^+ve^ cells in green (B, F, J, N, R, and V), total nuclei stained blue with DAPI (C, G, K, O, S, and W), and merged images (D, H, L, P, T, and X). The smaller boxes (D, H, L, P, T, and X) are enhanced images shown to illustrate colocalization of TUNEL, CD31, and DAPI within a single vessel. Scale bar = 100 *μ*m. (b) Quantitative data suggest FGF-9 inhibits capillary apoptosis following MI in C57BL/6 and db/db mice. ^#^ *p* < 0.05 versus sham and ^*∗*^ *p* < 0.05 versus MI.

Representative photomicrographs demonstrating capillary apoptosis are shown in Figure 2(a) for C57BL/6 (A–L) and db/db (M–X) mice with TUNEL positive nuclei in red (A, E, I, M, Q, and U), CD31 in green (B, F, J, N, R, and V), total nuclei stained blue with DAPI (C, G, K, O, S, and W), and merged images (D, H, L, P, T, and X). The smaller boxes (D, H, L, P, T, and X) are enhanced images shown to illustrate colocalization of TUNEL, CD31, and DAPI within a single vessel. Endothelial cell death, as evidenced by TUNEL and CD31 positive cells, was significantly elevated in C57BL/6 and db/db mice post-MI (*p* < 0.05, Figure 2(b)). However, treatment with FGF-9 dramatically abrogated capillary apoptosis in post-MI nondiabetic and diabetic mice (*p* < 0.05, Figure 2(b)). Moreover, endothelial cell death results were not significantly different between C57BL/6 and db/db mice within the same treatment group (Figure 2(b)).

### 3.3. FGF-9 Promotes c-Kit Activation and VSM Differentiation in Post-MI C57BL/6 and db/db Mice

**Figure 3:**
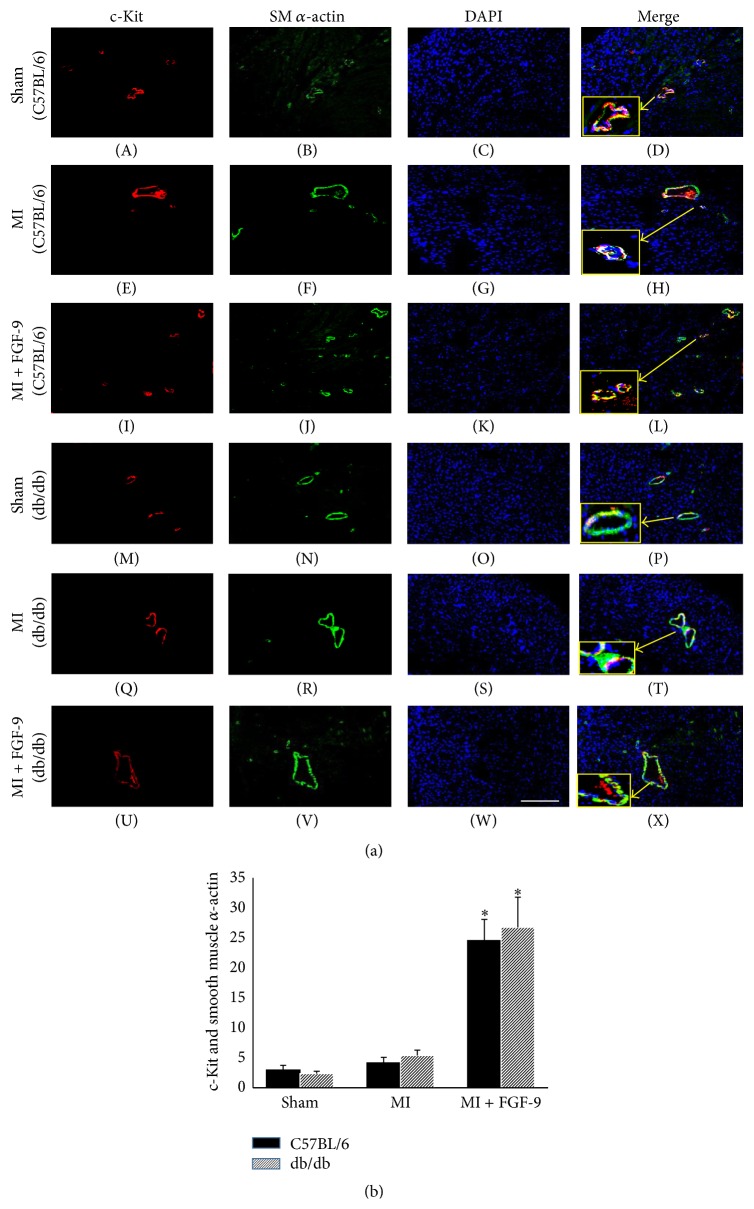
FGF-9 enhances c-Kit activation and vascular smooth muscle cell differentiation in post-MI C57BL/6 and db/db mice. Representative photomicrographs in (a) depict c-Kit^+ve^ progenitor cells in red (A, E, I, M, Q, and U), SM *α*-actin VSM cells in green (B, F, J, N, R, and V), total nuclei in blue (C, G, K, O, S, and W), and merged images (D, H, L, P, T, and X) for C57BL/6 and db/db groups. Enlarged images depicting colocalization of c-Kit^+ve^ and SM *α*-actin^+ve^ cells are presented in yellow boxes in (a) (D, H, L, P, T, and X). Scale bar = 100 *μ*m. (b) The number of c-Kit^+ve^/SM *α*-actin^+ve^ cells is significantly enhanced in the MI + FGF-9 group compared to the MI group in both C57BL/6 and db/db infarcted hearts. ^*∗*^ *p* < 0.05 versus MI. VSM = vascular smooth muscle.

The effect of FGF-9 on endogenous cardiac c-Kit^+ve^ progenitor cell activation and vascular smooth muscle cell differentiation was examined for all control and experimental animal groups. Representative images in Figure 3(a) demonstrate c-Kit^+ve^ progenitor cells in red (A, E, I, M, Q, and U), SM *α*-actin VSM cells in green (B, F, J, N, R, and V), total nuclei in blue (C, G, K, O, S, and W), and merged images (D, H, L, P, T, and X) for C57BL/6 and db/db groups. Enlarged images depicting colocalization of c-Kit^+ve^ and SM *α*-actin^+ve^ cells are presented in yellow boxes in Figure 3(a) (D, H, L, P, T, and X). Following MI, c-kit activation and VSM cell differentiation were not significantly different compared to the control group in both nondiabetic and diabetic mice (Figure 3(b)). However, when additionally treated with FGF-9 post-MI, the number of c-Kit^+ve^/SM *α*-actin^+ve^ cells was significantly enhanced in C57BL/6 and db/db mice compared to their MI-alone counterparts (*p* < 0.05, Figure 3(b)).

### 3.4. FGF-9 Enhances c-Kit Activation and EC Differentiation in Infarcted Myocardium

**Figure 4:**
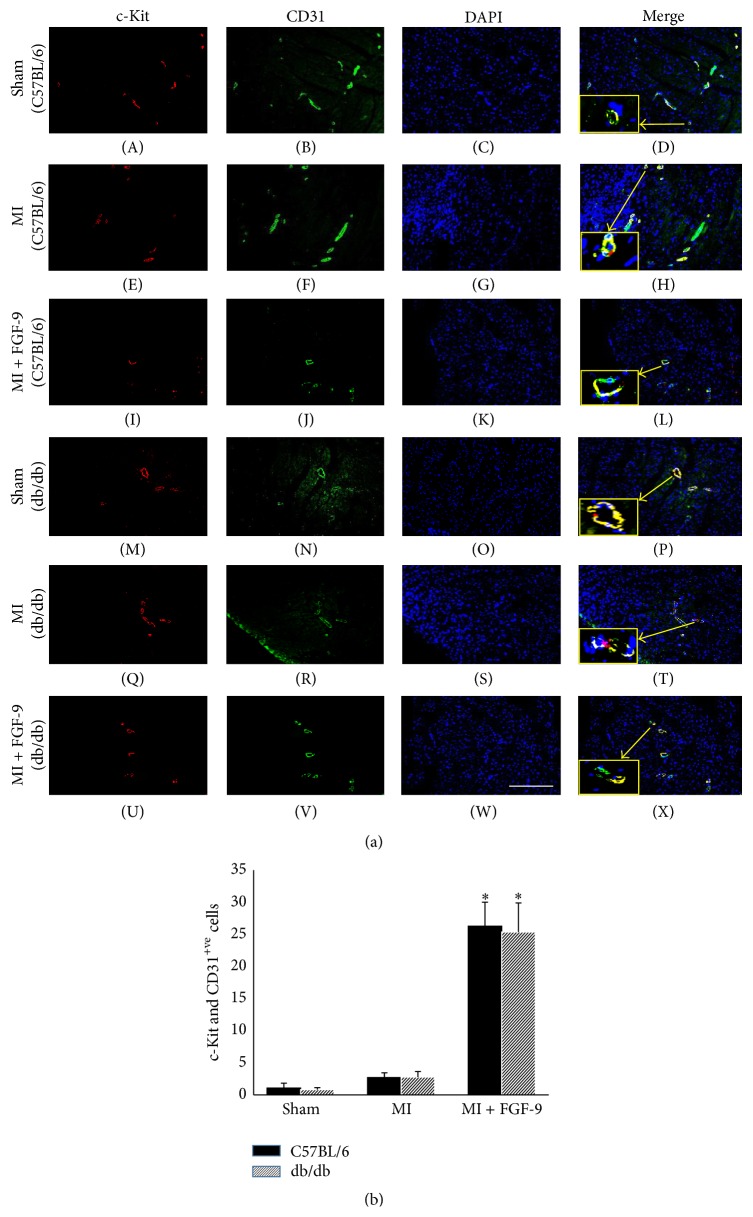
FGF-9 promotes c-Kit activation and endothelial cell differentiation in post-MI C57BL/6 and db/db mice. Representative images are shown in (a) demonstrating c-Kit^+ve^ cells in red (A, E, I, M, Q, and U), CD31^+ve^ cells in green (B, F, J, N, R, and V), total nuclei in blue (C, G, K, O, S, and W), and merged images (D, H, L, P, T, and X). Enlarged images depicting colocalization of c-Kit^+ve^ and CD31^+ve^ cells are presented in yellow boxes in (a) (D, H, L, P, T, and X). Scale bar = 100 *μ*m (b) Histogram of quantified c-Kit^+ve^/CD31^+ve^ cells in C57BL/6 and db/db infarcted hearts suggest FGF-9 enhances c-Kit activation and EC differentiation. ^*∗*^ *p* < 0.05 versus MI. EC = endothelial cell.

To assess the effects of FGF-9 on c-kit activation and endothelial cell differentiation in infarcted nondiabetic and diabetic myocardium, sections were double labeled with c-Kit and CD31. Representative images are shown in Figure 4(a) demonstrating c-Kit^+ve^ cells in red (A, E, I, M, Q, and U), CD31^+ve^ cells in green (B, F, J, N, R, and V), total nuclei in blue (C, G, K, O, S, and W), and merged images (D, H, L, P, T, and X). The number of cells positive for c-Kit and CD31 in heart sections from each group was quantified and our data suggests that progenitor cell activation and endothelial cell differentiation were unchanged following MI in C57BL/6 and db/db myocardium compared to sham counterparts (Figure 4(b)). Moreover, FGF-9 treated post-MI hearts show an increase in number of c-Kit^+ve^/CD31^+ve^ cells was significantly enhanced in both nondiabetic and diabetic mice compared to sham operated mice (*p* < 0.05, Figure 4(b)). Notably, the outcome of FGF-9 administration on c-Kit activation and EC differentiation was similar between C57BL/6 and db/db mice (Figure 4(b)). Collectively, our data implies FGF-9 treatment promotes endogenous c-Kit progenitor cell activation and differentiation into VSM cells and ECs in nondiabetic and diabetic infarcted myocardium.

### 3.5. Transplanted FGF-9 Promotes Vessel Formation in C57BL/6 and db/db Mice Post-MI

**Figure 5:**
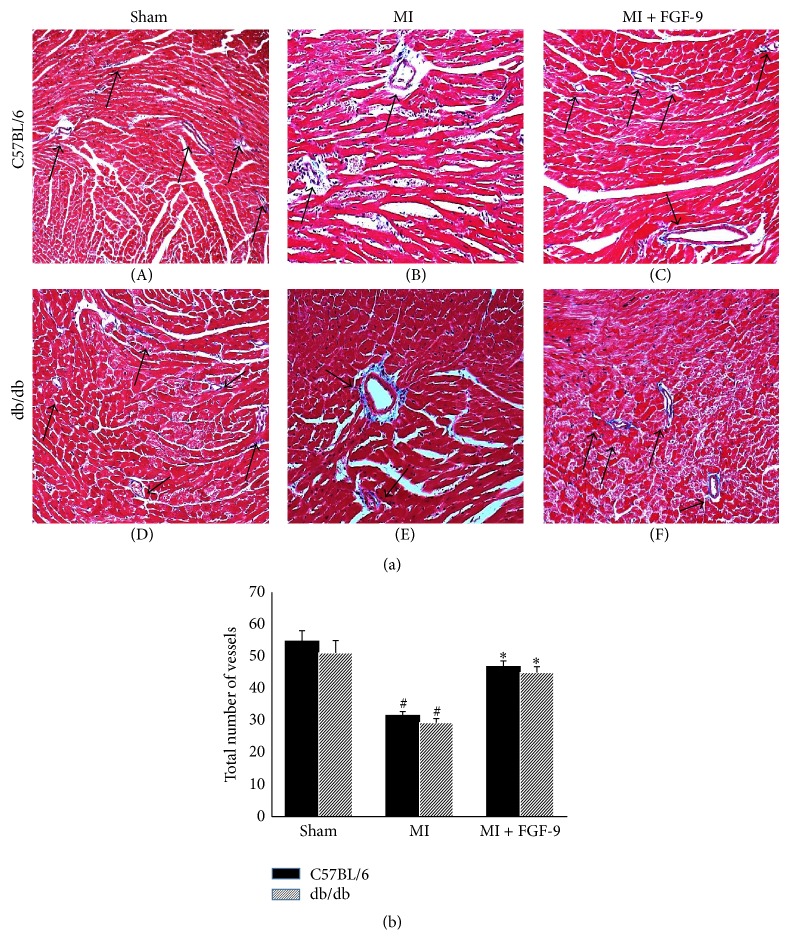
FGF-9 enhances vessel formation in postinfarcted nondiabetic and diabetic hearts. (a) Representative Masson's trichrome stained myocardial images for all control and experimental groups depicting blood vessels as indicated by the black arrows. (b) Data suggest that, following MI, the total number of vessels in the infarcted heart is significantly decreased whereas, following FGF-9 treatment, the total number of vessels is significantly increased relative to the MI group for both C57BL/6 and db/db mice. ^#^ *p* < 0.05 versus sham and ^*∗*^ *p* < 0.05versus MI.

To determine the effects of FGF-9 administration on coronary artery formation, the total number of small, medium, and large vessels was counted from Masson's trichrome stained heart sections as depicted in Figure 5(a). In both nondiabetic and diabetic myocardium, the number of total vessels was significantly decreased following MI compared to the sham groups (*p* < 0.05, Figure 5(b)). However, following FGF-9 treatment, a significant increase in the total number of myocardial vessels was significantly enhanced in the MI + FGF-9 group compared with the MI group in C57BL/6 and db/db mice (*p* < 0.05, Figure 5(b)).

### 3.6. FGF-9 Improved Left Ventricular Cardiac Function Post-MI in C57BL/6 and db/db Mice

**Figure 6:**
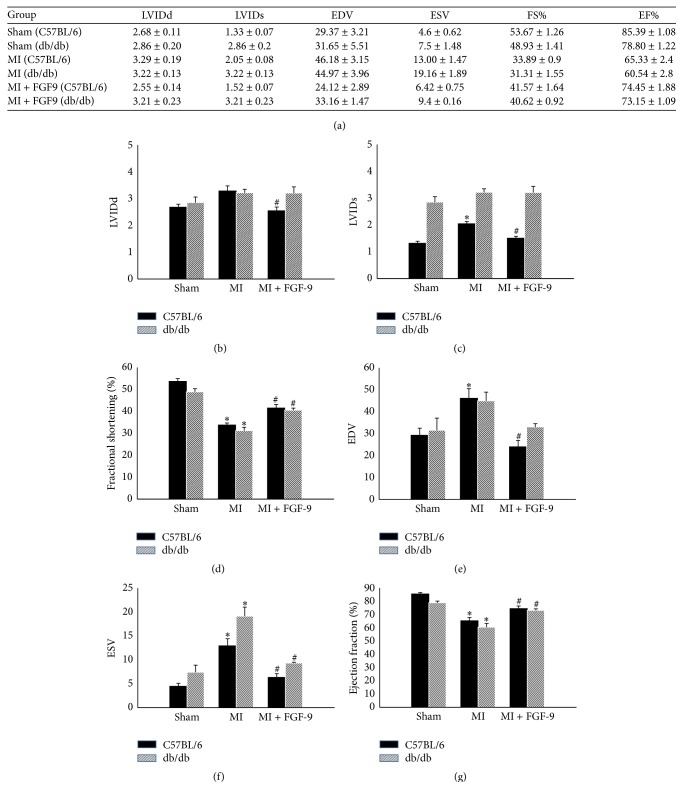
FGF-9 enhances left ventricular function in post-MI C57BL/6 and db/db mice. Two weeks following MI or sham surgery, heart function was assessed using transthoracic 2D echocardiography. (a) Table includes calculated cardiac functional data for all control and experimental groups. Histograms demonstrate quantitative analysis of (b) LVIDd, (c) LVIDs, (d) % fractional shortening, (e) EDV, (f) ESV, and (g) % ejection fraction. LVIDd = left ventricular internal dimension-diastole, LVIDs = left ventricular internal dimension-systole, FS = fractional shortening, EDV = left ventricular volume at end diastole, ESV = left ventricular volume at end systole, and EF = ejection fraction. ^#^ *p* < 0.05 versus sham and ^*∗*^ *p* < 0.05 versus MI.

Cardiac function was assessed two weeks after coronary artery ligation or sham operations via echocardiography for all control and experimental animals. All raw and calculated cardiac functional data are presented in Figures 6(a)–6(g). Following MI, left ventricular output, as indicated by fractional shortening and ejection fraction, was significantly hindered following MI surgery relative to sham controls in both C57BL/6 and db/db mice (*p* < 0.05, Figures 6(d) and 6(g), resp.). Importantly, a significant increase in left ventricular function was observed following FGF-9 administration in nondiabetic and diabetic mice relative to their MI group counterparts (*p* < 0.05, Figures 6(d) and 6(g)).

## 4. Discussion

After MI, myocardial angiogenesis is a reparative process responsible for resupplying ischemic tissue with oxygen and nutrients and is ultimately a key determinant of infarction and pathophysiological disease progression [6, 24]. Well documented, NIDDM is associated with an increased risk for developing secondary cardiovascular complications including coronary arterial disease (CAD), stroke, and peripheral arterial disease (PAD), all of which involve impaired angiogenesis [25–28]. Previous data have suggested that maladaptive myocardial angiogenesis in the context of diabetic patients is consequent, in part, to disrupted angiopoietins/Tie-2 signaling as well as diminished vascular endothelial growth factor (VEGF) expression [8–10]. Attempts to improve neoangiogenic outcomes post-MI have become an innovative target for many investigators using various proteins/small molecules including Apelin (APLN), thioredoxin-interacting protein (TXNIP) inhibitors, and peroxisome proliferator-activated receptors (PPARs) agonists, as well as various stem cell types [8, 29–34]. Notably, previously reported data have suggested that FGF-9 plays a role in coronary neovasculogenesis as well as enhancing microvessel density in the post-MI heart [13, 19]. However, there is no available data with regards to FGF-9 and angiogenesis in the postinfarcted diabetic heart nor has any attempt been made to elucidate mechanisms by which FGF-9 enhances post-MI angiogenesis, including the involvement of c-Kit stem cell activation. In the present study, we have evaluated the effects of FGF-9 treatment on vessel apoptosis, c-Kit^+ve^ stem cell stimulation, new vessel formation, and cardiac function in post-MI nondiabetic and diabetic mice. To the best of our knowledge, this is the first investigation into the vascular impact propagated by FGF-9 in the post-MI NIDDM injured myocardium.

To elucidate the impact of FGF-9 on the postischemic revascularization process, we first wanted to assess the ability of FGF-9 to inhibit post-MI vascular apoptosis. Widely accepted, vascular apoptosis plays a pivotal role in the manifestation and progression of a myriad of post-MI cardiac adverse outcomes but has yet to be investigated with regards to FGF-9. As supported by many other investigations, vascular apoptosis was significantly enhanced in both nondiabetic and diabetic mice post-MI [1, 6, 35]. Importantly, treatment with FGF-9 post-MI significantly abrogated vasculature apoptosis. Although this is the first report indicating inhibited vasculature apoptosis in the post-MI heart via FGF-9, supporting evidence of cellular protection has been identified in other cell types including neurons and fetal gonocytes [36, 37]. However, future studies are warranted to identify mechanisms by which FGF-9 provides vascular protection in the ischemic myocardium.

Evidence provided has suggested that neovascularization is a complex, multifactorial process involving proangiogenic growth factors including VEGF, granulocyte colony stimulating factor (G-CSF), insulin growth factor-1 (IGF-1), and stem cell factor (SCF) which in turn activate c-Kit stem cells and promote their differentiation into VSM cells and ECs [6]. With this aforementioned data in mind, we examined whether FGF-9 had any impact on c-Kit positive stem cell activation in the nondiabetic and diabetic infarcted heart. Importantly, a significant increase in c-Kit^+ve^ progenitor cells costained with SM *α*-actin, identifying VSM cells, and c-Kit^+ve^ progenitor cells costained with CD31, identifying ECs, were identified following post-MI FGF-9 treatment. Of note, there was a slight increase in the amount of c-Kit^+ve^/SM *α*-actin^+ve^ cells and c-Kit^+ve^/CD31^+ve^ cells in the MI group compared with sham, but this trend was not statistically significant. Although the exact reasons for this increase are unknown, we postulate that it may be consequent to post-MI microenvironmental alterations in cytokine/growth factor expression. Additionally, nondiabetic and diabetic mice had similar results regarding concentrations of c-Kit^+ve^/SM *α*-actin^+ve^ cells and c-Kit^+ve^/CD31^+ve^ cells in the infarcted heart. We suggest FGF-9 may promote neovascularization by inhibiting apoptosis and by acting directly on c-kit cells or enhancing proangiogenic factors that are disrupted in both nondiabetic and diabetic post-MI hearts. Altogether, our quantitative data suggest that endogenous c-Kit progenitor cells were activated and differentiated into neovascular cell types in the presence of FGF-9 in the infarcted heart. In this paper, we report a correlation between FGF-9 and c-Kit progenitor cell activation; however, future studies are necessitated to identify exact signaling pathways and cytokine/chemokine mediators, which drive FGF-9-induced neovascularization.

To verify enhanced neovascularization in the C57BL/6 and db/db infarcted hearts, sections were stained with Masson's trichrome and the total numbers of vessels were counted for all control and experimental groups. Quantitative data suggest that there was significant increase in the total number of myocardial vessels following post-MI FGF-9 treatment compared to the MI group counterparts. Our data further corroborate our earlier findings of enhanced c-Kit progenitor cell activation and VSM cell and EC differentiation in the MI + FGF-9 group. Supportive data previously published demonstrate the ability of multiple stem cells (embryonic stem (ES) cells, mesenchymal stem cells (MSCs), and induced pluripotent stem (iPS) cells), to promote c-Kit progenitor cell activation and ultimately enhance angiogenesis and neovascularization in post-MI animal models [6, 22, 38–41].

Finally, we assessed the impact of neovascularization on the overall left ventricular output post-MI for all C57BL/6 and db/db groups. Two weeks after MI, cardiac function was obtained by transthoracic 2D echocardiography. Functional data, including fractional shortening and ejection fraction, were significantly improved following FGF-9 treatment in infarcted nondiabetic and diabetic mice relative to MI counterparts. Collectively, our data suggests that FGF-9 inhibits vascular apoptosis and enhances angiogenesis, which contributes to an improvement in cardiac function following MI.

In conclusion, we report for the first time that FGF-9 prevents post-MI vascular apoptosis, enhances c-Kit activation and their differentiation into VSM cells and ECs, and improves overall cardiac function in C57BL/6 and db/db mice. Our study provides evidence that FGF-9 enhances angiogenesis in infarcted myocardium and may have therapeutic potential for post-MI nondiabetic and diabetic patients. Moreover, we suggest that mechanisms of enhanced angiogenesis may deviate between diabetic and nondiabetic subjects and future studies are warranted to evaluate these differences including infarct size and augmented molecular signaling pathways as a result of FGF9 treatment.
