# Corrigendum to “Fibroblast Growth Factor-9 Activates c-Kit Progenitor Cells and Enhances Angiogenesis in the Infarcted Diabetic Heart”

**DOI:** 10.1155/2018/6985498

**Published:** 2018-08-19

**Authors:** Dinender K. Singla, Jing Wang

**Affiliations:** Biomolecular Science Center, Burnett School of Biomedical Sciences, College of Medicine, University of Central Florida, Orlando, FL 32816, USA

In the article titled “Fibroblast Growth Factor-9 Activates c-Kit Progenitor Cells and Enhances Angiogenesis in the Infarcted Diabetic Heart” [1], the name of the first author was given incorrectly as Dinender Singla. The author's name should have been written as Dinender K. Singla. The revised authors' list is shown above.

## References

[1] Singla D., Wang J. (2016). Fibroblast growth factor-9 activates c-kit progenitor cells and enhances angiogenesis in the infarcted diabetic heart. *Oxidative Medicine and Cellular Longevity*.

